# Unfinished nursing care in four central European countries

**DOI:** 10.1111/jonm.12896

**Published:** 2019-11-26

**Authors:** Renáta Zeleníková, Elena Gurková, Adriano Friganovic, Izabella Uchmanowicz, Darja Jarošová, Katarína Žiaková, Ilona Plevová, Evridiki Papastavrou

**Affiliations:** ^1^ Department of Nursing and Midwifery Faculty of Medicine University of Ostrava Ostrava Czech Republic; ^2^ Department of Nursing Faculty of Health Sciences Palacky University in Olomouc Olomouc Czech Republic; ^3^ University Hospital Zagreb Zagreb Croatia; ^4^ University of Applied Health Sciences Zagreb Croatia; ^5^ Department of Clinical Nursing Faculty of Health Sciences Wroclaw Medical University Wroclaw Poland; ^6^ Department of Nursing Jessenius Faculty of Medicine in Martin Comenius University in Bratislava Martin Slovakia; ^7^ Department of Nursing School of Health Sciences Cyprus University of Technology Limassol Cyprus

**Keywords:** hospital nurses, PIRNCA, survey, unfinished nursing care

## Abstract

**Aim:**

The main aim of the research was to describe and compare unfinished nursing care in selected European countries.

**Background:**

The high prevalence of unfinished nursing care reported in recently published studies, as well as its connection to negative effects on nurse and patient outcomes, has made unfinished care an important phenomenon and a quality indicator for nursing activities.

**Methods:**

A cross‐sectional descriptive study was undertaken. Unfinished nursing care was measured using the Perceived Implicit Rationing of Nursing Care questionnaire (PIRNCA). The sample included 1,353 nurses from four European countries (Croatia, the Czech Republic, Poland and Slovakia).

**Results:**

The percentage of nurses leaving one or more nursing activities unfinished ranged from 95.2% (Slovakia) to 97.8% (Czech Republic). Mean item scores on the 31 items of the PIRNCA in the total sample ranged from 1.13 to 1.92. Unfinished care was significantly associated with the type of hospital and quality of care.

**Conclusion:**

The research results confirmed the prevalence of unfinished nursing care in the countries surveyed.

**Implications for Nursing Management:**

The results are a useful tool for enabling nurse managers to look deeper into nurse staffing and other organizational issues that may influence patient safety and quality of care.

## 1 INTRODUCTION

The number of scientific papers related to unfinished nursing care has increased significantly over the last decade, with several extensive reviews concluding that unfinished nursing care is a problem worldwide (Jones, Hamilton, & Murry, 2015; Papastavrou, Andreou, & Efstathiou, 2014b). Research into unfinished care has increased interest in the phenomenon since it was first recognized under the term “nursing care left undone” in Canada, England, Germany, the United States and Scotland (Aiken et al., 2001). In Switzerland, the concept has been introduced as “implicit care rationing” (Schubert et al., 2008). Several other terms have been used in Europe, for instance, “bedside rationing” and “care left undone” (Ausserhofer et al., 2014; Papastavrou, Andreou, Tsangari, & Merkouris, 2014a). For this study, we chose the term “unfinished nursing care” as an umbrella term (Jones et al., 2015).

The phenomenon is recognized by the Agency for Healthcare Research and Quality as a global risk affecting healthcare organizations all over the world (AHRQ, 2019). Despite differences in healthcare and funding systems, the problem of unfinished nursing care has been identified in a number of European countries. Findings from Ausserhofer et al. (2014) confirm that European nurses choose to perform certain nursing interventions and to withhold others.

The concept of unfinished nursing care is relatively new in Central Europe. Although there has been an increasing number of studies on the subject in international journals over the last decade, there is a dearth of complex information on the issue in Central European countries. For this study, four countries were chosen: Croatia, the Czech Republic, Poland and Slovakia, all post‐communist central European countries. Nurse shortages, raised migration of nurses, low salaries and an ageing workforce as well as increasing outflow of nurses out of the health system are the main concerns across these four countries (Marć, Bartosiewicz, Burzyńska, Chmiel, & Januszewicz, 2019). In addition, in the Czech Republic and Slovakia the tendency of healthcare providers to substitute registered nurses with practical nurses may influence the provided nursing care.

According to OECD statistics (OECD, 2019), there are eight nurses/per 1,000 people in the Czech Republic, 5.2 nurses/per 1,000 people in Poland and 5.7 nurses/per 1,000 people in Slovakia (OECD, 2019). In Croatia, there are 5.8 nurses/1,000 inhabitants (OECD, 2017). All have fewer nurses than the EU average (8.4). There are serious concerns about possible decreases in these numbers over the coming years.

When these countries entered the European Union (EU), they had to contend with nursing education reforms in accordance with the EU requirements. Before the EU accession, nurses from the central and eastern European countries had been educated in secondary vocational training (after 8‐year primary school education) at specialized high schools (Kalauz, Orlic‐Sumić, & Simunec, 2008; Simunovic et al., 2010; Ślusarska, Zarzycka, Dobrowolska, Marcinowicz, & Nowicki, 2018; Tóthová & Sedláková, 2008). Secondary vocational nursing schools in the Czech Republic and Slovakia no longer offer the general nurse study programme. However, some nurses trained in these schools before 2004 in Czech Republic and Slovakia still work at hospitals, as general nurses. Today, there are still students who graduate from secondary nursing schools, but they are trained as “practical nurses” with lower competencies than general nurses. These days qualified nurses are required to have a bachelor degree at university or diploma at higher education institutions in all four surveyed countries (after 12 years of general education). In Poland, since 2004 bridging studies—intended for nurses graduating from secondary medical schools/colleges—have become very popular as a supplementary to bachelor degrees (Ślusarska et al., 2018). A dual entry system operates in Croatia, with nursing students able to become nurses after completing vocational school (general care nurse), or a bachelor degree at university (Kalauz et al., 2008). The present status of Croatian nursing education is contentious (Simunovic et al., 2010), and study at vocational school is for 5 years: 2 years of general education and 3 years of nursing (Simunovic et al., 2010). Croatian nurses' competencies after vocational education and bachelor degree programme are almost the same which create confusion in nurses' practice and lead to dissatisfaction (Simunovic et al., 2010).

## 2 BACKGROUND

There are a number of published conceptual analyses and conceptual frameworks related to the concept of rationed/missed/unfinished nursing care (Bail & Grealish, 2016; Hessels, Flynn, Cimiotti, Cadmus, Gershon, 2015; Kalisch, Landstrom, & Hinshaw, 2009; Lucero, Lake, & Aiken, 2009; Schubert, Glass, Clarke, Schaffert‐Witvliet, & De Geest, 2007). For this study, the conceptual framework of unfinished nursing care described by Jones et al. (2015) and Jones, Willis, Amorim‐Lopes, Drach‐Zahavy, RANCARE Consortium COST, (2019) was chosen. Unfinished care in this conceptual model is a component of the process of care between organizational system structures and outcomes/effects. Organizational variables, nursing work environment, care philosophy, and nurse and patient variables are considered as antecedents (Jones et al., 2015). They are also factors that influence clinical judgment, decision‐making, setting of priorities and triage processes in nursing practice (Schubert et al., 2013, 2007). The model suggests that unfinished care is directly related to patient and nurse outcomes. In this study, we wanted to explore associations between unfinished care and selected nurse outcomes (i.e. job satisfaction and intention to leave) and patient outcome (nurse‐assessed quality of care). The results of the RN4CAST study involving 12 European countries (Belgium, England, Finland, Germany, Greece, Ireland, the Netherlands, Norway, Poland, Spain, Sweden and Switzerland) indicate that all these countries have had to deal with the problem of nursing care quality, patient safety, job satisfaction and burnout (Aiken et al., 2012). According to Ausserhofer et al. (2014), unfinished nursing care might play an important role in nurse outcomes (job satisfaction, burnout and intention to leave). Unfinished nursing care has previously been linked to a decrease in job satisfaction (Bekker, Coetzee, Klopper, & Ellis, 2015; Jones, 2014; Kalisch, Tschannen, & Lee, 2011) and an increase in nurse intention to leave (Tschannen, Kalisch, & Lee, 2010). In addition, the frequency of unfinished care is increasingly being considered an indicator in the assessment of quality of care (Recio‐Saucedo et al., 2018). Associations between unfinished care and overall quality of care have been documented before (Ball, Murrells, Rafferty, Morrow, & Griffiths, 2014; Jones, 2014; Sochalski, 2004; Zúñiga et al., 2015).

## 3 AIM

The main aim of the research was to describe and compare unfinished nursing care in four Central European countries (Croatia, the Czech Republic, Poland and Slovakia). A secondary aim was to explore the relationships between selected variables and unfinished nursing care.

## 4 METHODS

### 4.1 Design

A cross‐sectional descriptive study was undertaken.

### 4.2 Sample

The research sample included 1,353 nurses from four European countries (306 nurses from the Czech Republic, 356 nurses from Slovakia, 253 nurses from Poland and 438 nurses from Croatia). The target population was nurses from acute care departments employed in acute care hospitals in the public or private sector in Central European countries (Croatia, the Czech Republic, Poland and Slovakia). Only fully qualified nurses were included in the survey. Inclusion criteria were nurses from acute care departments with at least 1 year of experience; nurses engaged in direct patient care; and nurses with an understanding of the national languages. Exclusion criteria were nurses in managerial positions, home care nurses and practical nurses (formerly nurse assistants).

Since the population of nurses is large (around 270,000 nurses in Poland, 90,000 nurses in Czech Republic, approximately 40,000 nurses in Croatia and in Slovakia), we set the sample size at a minimum of 196 nurses from each country. This sample gives the study a margin of error of ±7% (confidence interval 95%) in determining the prevalence of unfinished nursing care. Online sample size calculator (Qualtrics^®^) was used.

In Slovakia, the questionnaires were distributed to all nurses from 16 selected departments of eight acute care hospitals (six private and two public) with at least 100 beds. The response rate for Slovak nurses was 82.38%. In the Czech Republic, the questionnaires were distributed to nurses from 26 selected departments of eight hospitals (three private and five public) with more than 100 beds. The response rate for Czech nurses was 70.18%. In Croatia, the sample consisted of nurses working in four university hospitals. The questionnaires were distributed to nurses from 17 selected departments. The response rate for Croatian nurses was 73%. In Poland, the sample consisted of nurses from a university hospital. The response rate for Polish nurses was 79%.

### 4.3 Data collection

Data collection was carried out from April 2018 to November 2018. A pencil and paper questionnaire was administered at a single point in time. The questionnaires were distributed to hospital nurses during day shifts by a team coordinated by the principal researchers in each country. Nurses had three‐four weeks to complete the questionnaires.

### 4.4 Research instruments

For the purpose of the study, the following instruments were used: demographic data sheet; the PIRNCA questionnaire (Jones, 2014); and questions regarding job satisfaction, intention to leave and overall quality of care.

Demographic data included: personal (age, education, specialisation); employment (unit, professional experience, work experience in current workplace, work hours, absent days or shifts, hours of overtime, perceived adequacy of staffing, leaving intentions—the last five items were adopted from the MISSCARE Survey (Kalisch et al., 2009)); and organizational variables (hospital size and type).

#### 4.4.1 Unfinished nursing care

Unfinished nursing care is “a problem of time scarcity that precipitates the process of implicit rationing through clinical priority setting among nursing staff resulting in the outcome of care left undone” (Jones et al., 2015).

Unfinished nursing care was measured using the PIRNCA questionnaire (Perceived Implicit Rationing of Nursing Care), an inventory of 31 nursing activities (Jones, 2014). Nurses were asked to rate how often they were unable to complete each of the 31 activities for patients on their previous seven shifts: “never” = 1, “rarely” = 2, “sometimes” = 3 and “often” = 4 (Van Fosson, 2017). In the original study (Jones, 2014), the reliability of the PIRNCA was high (0.97).

In our study, the PIRNCA was scored in two ways: a count of dichotomized occurrences for a specific cut‐off point (percentage of nurse rationing greater than “never”) and the arithmetic mean score across all inventory items (a mean composite score; Jones, Gemenihardt, Thomson, & Hamilton, 2016; Van Fosson, 2017).

#### 4.4.2 Overall job satisfaction

Nurses’ job satisfaction is “the nurses’ positive feeling response to the work conditions that meet his or her desired needs as the result of their evaluation of the value or equity in their work experience” (Liu, Aungsuroch, & Yunibhand, 2015).

Overall job satisfaction (OJS) was assessed on an 11‐point scale (0—“It is terrible” to 10—“I love it”), using the single item: “Considering all aspects of your job, as well as your own values, ideals and goals, how satisfied are you with your current nursing job?” (Kramer & Schmalenberg, 2004; Schmalenberg & Kramer, 2008).

#### 4.4.3 Nurse‐assessed quality of patient care on unit

Quality of care is “the degree to which health services for individuals and populations increase the likelihood of desired health outcomes and are consistent with current professional knowledge” (Institute of Medicine, 1990).

Overall quality of patient care experience was measured on an 11‐point scale (0—“dangerously low” to 10—“very high quality”) using the single item (Kramer & Schmalenberg, 2004; Schmalenberg & Kramer, 2008): “Circle the number that indicates the usual quality of care provided to patients on your unit.”

#### 4.4.4 Intention to leave

Intention to leave is anticipation to leave the current position of nurse (Tschannen et al., 2010).

Nurses’ intention to leave their organization was measured according to the study by Yamaguchi, Inoue, Harada, and Oike (2016). Respondents were asked one question: “How do you see your working life in the future?” Response options were on a scale from 1 to 4 (1 = “I would like to continue working at my current workplace”, 4 = “I would like to change my unit/organization”).

Participants’ intention to leave their profession was measured using one item: “How do you see your career as a nurse in the future?” Response options were on a scale from 1 to 4 (1 = “I want to continue working as a nurse”, 4 = “I don’t want to continue working as a nurse”), with a higher score indicating a stronger intention to leave the profession.

#### 4.4.5 Translation and validation of the PIRNCA

Permission to translate and use the PIRNCA questionnaire was obtained from the author of the original questionnaire (Jones, 2014) prior to data collection. The PIRNCA instrument was translated from English into the four national languages (Croatian, Czech, Polish and Slovak) using forward–backward translation.

However, psychometric procedures for each language version (Czech, Slovak, Polish and Croatian) included reliability analysis and construct validity evaluation. The final versions of each language version had to be tested by the same procedures that had been used on the original instrument (Jones, 2014). The structure of each language version was studied using exploratory factor analysis (EFA), since the PIRNCA is a one‐dimensional inventory. For factor extraction and interpretation of factor loadings, principal component analysis (PCA) with varimax rotation was applied. First, the data were subjected to unforced factor analysis. Second, EFA with forced one‐factor solution was performed, since it was used in the original US sample (Jones, 2014). Factorability of each version was tested by the correlation matrix, communalities, Kaiser–Meyer–Olkin (KMO) measure and Bartlett's sphericity test (Jones, 2014). All assumptions for the performance of the EFA were met, and therefore, PCA with pairwise exclusion for missing data was applied for factor extraction and interpretation of factor loadings and assessment of the dimensionality of each version. Evidence of concurrent validity of each version was provided by correlation analysis with two related constructs (Jones, 2014): overall job satisfaction and overall work experience. For examining the associations between variables, parametric Pearson's correlations were performed. Regarding reliability of the instrument, internal consistency was tested by Cronbach's alpha (*α*_c_). A *p*‐value <.05 was taken to indicate statistical significance for all comparisons.

Unforced factor analysis on the PIRNCA generated six factors with eigenvalues >1 in the Czech version; four factors in the Polish and the Slovak versions; and five factors in the Croatian version. A six‐factor solution explained 64.56% of variance in the Czech version; four‐factor solutions explained 73.79% of variance in the Polish version and 61.04% in the Slovak version; five‐factor solutions explained 69.17% of the variance in the Croatian version. Variance extracted by factor 1 was the highest in all language versions (the value was 4.51 after rotation in the Czech version; 6.69 in the Polish version; 5.07 in the Slovak version and 5.25 in the Croatian version), and this factor also explained the greatest part of variance in each version (14.54% after rotation in the Czech version; 21.58 in the Polish version; 16.32 in the Slovak version and 16.92 in the Croatian version). Factor loading of the items in factor 1 was in a range between 0.43 and 0.70 in the unrotated unforced analyses of the Czech version; between 0.57 and 0.87 in the Polish version; and between 0.50 and 0.79 in the Croatian and Slovak versions. The original factors were not interpretable in each version, and the scree plots did not really support multiple factors.

The forced one‐factor solution explained only 39.64% of the variance in the Czech version; 59.72% in the Polish version; 44.52% in the Slovak version and 48.81% in the Croatian version. All 31 items had factor loadings exceeding 0.50 in each language version. The results of EFA showed that all 31 items loaded to one component, and factor loadings were strong in each language version.

### 4.5 Concurrent validity and relations with other variables

In the Czech version, negative non‐significant correlations were noted between the PIRNCA composite score and both overall job satisfaction (*r* = −.03; *p* = .70) and quality of care (*r* = −.06; *p* = .38). Negative, moderate and significant correlations were found between the PIRNCA composite score and both overall job satisfaction (*r* = −0.46 in the Polish version; −0.37 in the Croatian version; −0.32 in the Slovak version) and quality of care (*r* = −0.57 in the Polish version; −0.43 in the Croatian and −0.35 in the Slovak version).

### 4.6 Data analysis

Descriptive statistics were reported for survey results. Cronbach's alpha internal consistency coefficient was determined for all language versions of the PIRNCA. Statistical analysis further involved group comparisons and correlation between different variables. Data analysis was performed without missing data. Variables assumed a normal distribution with skewness <1.00; parametric tests were therefore used. To test the associations between variables, parametric Pearsons’ correlations and multiple regression analyses were calculated. Statistical analysis of group comparisons was performed using multifactorial ANOVA and Fisher's least significant difference (LSD) procedure. Proportion comparisons were performed with the Pearson's chi‐square test. A *p*‐value of <.05 was set to indicate statistical significance. The Statistical Package for Social Sciences software (SPSS, Inc) was used for statistical analysis.

## 5 RESULTS

### 5.1 Sample characteristics

Table 1 presents the characteristics of the nurses participating in the study. Most respondents were female (94.7%), and more than one half of the nurses had at least a bachelor degree. The majority of the respondents reported working rotating shifts. Only 9.9% of the nurses reported staffing as adequate 100% of the time.

**Table 1:** Individual, employment and organizational variables

Variable	*N*	%
Country (*n* = 1,353)		
Czech Republic	306	22.6
Slovak Republic	356	26.3
Poland	253	18.7
Croatia	438	32.4
Highest nursing degree (*n* = 1,334)		
Secondary nursing school or diploma	643	47.8
Bachelor degree or higher	701	52.2
Postgraduate Education—specialized programmes for nurses	294	21.8
Work hours (*n* = 1,094)		
Rotating shifts	803	73.4
Hours of overtime in past 3 months (*n* = 1,097)		
None	307	28.0
1–12 hr	389	35.5
More than 12 hr	401	36.5
Hours worked per week (*n* = 1,343)		
30 hr or more	1,288	95.5
Days or shifts absent in past 3 months (*n* = 1,096)		
None – 1 day or shift	927	86.6
2 or more days or shifts	169	15.4
Perception of staffing adequacy (*n* = 1,340)		
100% of the time	132	9.9
75% of the time	321	24.0
50% of the time	365	27.1
25% or less of the time	522	39.0
Leaving intentions of current position (*n* = 1,088)		
in the next 6 months	51	4.7
in the next 1 year	149	13.7
no plans to leave	888	81.6
Type of hospitals (*n* = 1,091)		
University/faculty/teaching	508	46.6
General	553	53.4
Size of hospitals (*n* = 1,331)		
<300 beds	290	21.8
300–600 beds	373	28.0
>600 beds	668	50.2
Unit (*n* = 1,328)		
Medical units	249	18.8
Surgical units	482	36.3
ICU	276	20.3
Others	321	23.6
	**Mean**	***SD***
Age	38.61	10.52
Professional experience	15.91	10.87
Years of experience on current workplace	11.28	9.77

### 5.2 Frequencies and patterns of unfinished nursing care

Mean item scores of the 31 items in the total sample (Table 2) ranged from 1.13 to 1.92 (less than “rarely” to “rarely”). Patterns of unfinished care were analysed by dichotomized PIRNCA scoring (percentage of nurses with a frequency rating higher than never; Jones, 2014; Jones et al., 2015). Dichotomized scoring showed that a high percentage of nurses reported that they left one or more nursing care activities unfinished. The percentage of nurses leaving one or more care activities unfinished was high and ranged from 95.2% (Slovakia) to 97.8% (Czech Republic). About 97.7% of nurses in Poland and 97.5% of nurses in Croatia reported that they left one or more activities of care unfinished. On average, each nurse left 13.88 of care activities unfinished (on average Czech nurses left 9.66; Polish nurses 15.32; Slovak nurses 11.01 and Croatian nurses 17.45 of care activities unfinished).

**Table 2:** Elements of unfinished nursing care: comparison between four countries (one way ANOVA)†

Item	SK	CZ	PL	HR	Total	*p*
	Mean (*SD*)	Mean (*SD*)	Mean (*SD*)	Mean (*SD*)	Mean (*SD*)	
Documenting						
Document all nursing care/interventions	1.53 (0.96)	1.34 (0.70)	1.74 (1.10)	**2.08** (1.06)	1.71 (1.01)	.000
Document initiation or revision of a patient's plan of care	1.51 (0.95)	1.18 (0.70)	1.63 (1.10)	1.78 (1.00)	1.55 (0.97)	.000
Document assessments and monitoring activities	1.50 (0.97)	1.24 (0.69)	1.67 (1.12)	1.83 (1.01)	1.58 (0.98)	.000
Evaluating						
Evaluate plan of care	**1.58** (0.98)	1.28 (0.82)	1.83 (1.12)	**1.97** (1.05)	1.69 (1.03)	.000
Monitoring/Surveillance						
Monitoring patient physiological status	1.21 (0.74)	1.08 (0.75)	1.57 (1.13)	1.68 (0.89)	1.40 (0.91)	.000
Monitoring patient affect and behaviour	1.56 (0.93)	1.36 (1.00)	1.73 (1.18)	1.88 (0.97)	1.65 (1.02)	.000
Monitoring patient physical safety	1.31 (0.80)	1.14 (0.79)	1.67 (1.03)	1.75 (0.88)	1.48 (0.90)	.000
Follow‐up on change in patient status	1.42 (0.85)	1.27 (0.69)	1.67 (1.08)	1.82 (0.95)	1.56 (0.92)	.000
Review documentation by care team	**1.57** (0.98)	**1.40** (0.80)	1.77 (1.13)	**2.02** (0.97)	**1.72** (1.00)	.000
Supervise care						
Adequate supervision of delegated tasks	1.54 (0.90)	**1.54** (0.90)	1.85 (1.10)	1.76 (0.98)	1.67 (0.97)	.000
Hygiene						
Routine hygiene for patients	1.32 (0.84)	1.02 (0.76)	1.68 (1.24)	1.66 (1.01)	1.43 (1.00)	.000
Routine skin care	1.21 (0.78)	0.96 (0.72)	1.66 (1.15)	1.57 (0.95)	1.36 (0.94)	.000
Change soiled linen	1.47 (0.92)	1.07 (0.73)	1.74 (1.18)	**2.05** (1.11)	1.62 (1.07)	.000
Nutrition						
Assist with feeding patient	1.26 (0.80)	1.06 (0.74)	1.42 (1.14)	1.57 (0.89)	1.35 (0.91)	.000
Administer enteral or parenteral nutrition	1.06 (0.62)	0.79 (0.56)	1.20 (0.97)	1.38 (0.79)	1.13 (0.77)	.000
Physical comfort						
Implement measures to promote physical comfort	1.27 (0.79)	1.23 (0.87)	1.62 (1.10)	1.72 (0.89)	1.47 (0.93)	.000
Mobility						
Assist with needed ambulation	1.37 (0.90)	1.20 (0.85)	1.69 (1.26)	1.86 (1.02)	1.55 (1.04)	.000
Mobilize or change patient position	1.36 (0.86)	1.09 (0.75)	1.72 (1.16)	1.83 (0.98)	1.52 (0.98)	.000
Elimination						
Timely assistance with elimination	1.29 (0.74)	1.05 (0.74)	1.47 (1.06)	1.57 (0.87)	1.36 (0.87)	.000
Treatment, Tests, Procedures						
Administer medications	1.09 (0.60)	1.06 (0.45)	1.33 (0.82)	1.42 (0.77)	1.23 (0.70)	.000
Change intravenous access sites, tubing or dressing	1.15 (0.66)	1.11 (0.56)	1.44 (0.88)	1.54 (0.85)	1.33 (0.78)	.000
Provide wound care	1.16 (0.72)	0.90 (0.61)	1.31 (0.90)	1.54 (0.86)	1.25 (0.82)	.000
Prepare patients for treatments, tests…	1.19 (0.71)	1.11 (0.54)	1.89 (1.21)	1.73 (0.90)	1.48 (0.90)	.000
Adherence to safe patient handling guidelines	1.40 (0.92)	1.38 (0.82)	1.72 (1.27)	1.87 (0.99)	1.61 (1.02)	.000
Patient/family teaching						
Patient and family teaching	1.56 (0.89)	**1.45** (0.80)	**2.13** (1.31)	1.95 (0.97)	**1.77** (1.02)	.000
Emotional or psychological support						
Emotional or psychological support	**1.63** (0.97)	**1.63** (0.93)	**2.09** (1.27)	**2.25** (1.02)	**1.92** (1.08)	.000
Infection control						
Adhere to infection control guidelines	1.11 (0.62)	1.11 (0.56)	1.69 (1.10)	1.68 (0.88)	1.40 (0.85)	.000
Timeliness of care						
Timely response to request/need	**1.81** (1.01)	**1.80** (0.90)	**1.92** (1.13)	1.86 (1.06)	**1.85** (1.03)	.000
Coordination & Discharge Planning						
Important conversation with patient or family about discharge	1.48 (0.93)	1.27 (0.72)	**1.91** (1.06)	1.65 (1.03)	1.57 (0.97)	.000
Important conversation with team member	**1.61** (1.00)	1.36 (0.84)	**2.00** (1.17)	1.89 (1.00)	**1.72** (1.03)	.000
Important conversation with external agency	1.19 (1.07)	0.83 (1.00)	1.64 (1.26)	1.47 (1.16)	1.28 (1.16)	.000
PIRNCA score	1.38 (0.57)	1.28 (0.47)	1.70 (0.86)	1.76 (0.67)	1.56 (0.68)	.000
Cronbach's alpha of the PIRNCA	0.957	0.860	0.936	0.929	0.967	

^†^ Boldface text indicates the first five most frequently left unfinished activities in each country and in all sample.

Item‐level rationing frequencies ranged from 23.6% to 61.6% (Table 3). The areas of care most frequently left unfinished were consistent based on mean scale responses and dichotomized responses. The care interventions most frequently unfinished were identified as emotional or psychological support to a patient or family; timely response to requests; patient education; and important conversations with another member of a patient's multidisciplinary team (Tables 2 and 3). Significant differences in all 31 activities of the PIRNCA and in overall mean score were found between countries (Table 2). Considerable differences between participating countries were also observed in the percentage of nurses with a frequency higher than never (Table 3). Results of post hoc tests (Fisher's LSD procedure) revealed no differences in the composite mean PIRNCA scores between Slovak and Czech nurses (*p* = .09), and between Croatian and Polish nurses (*p* = .23). Slovak and Czech nurses reported less unfinished care than Polish and Croatian nurses (*p* = .000).

**Table 3:** Elements of unfinished nursing care: comparison between four countries (Pearson's chi‐square test)†

Item	SK (%)		CZ (%)		PL (%)		HR (%)		Total (%)		*p*
	Never or No needed	>Never	Never or No needed	>Never	Neve or No needed	>Never	Never or No needed	>Never	Never or No needed	>Never	
Documenting											
Document all nursing care/interventions	15.4	11.4	15.9	6.5	8.9	9.1	10.8	22.1	51.0	**49.0**	.000
Document initiation or revision of a patient's plan of care	15.7	11.1	17.0	5.3	10.1	7.9	13.7	19.3	56.4	**43.6**	.000
Document assessments and monitoring activities	15.7	11.1	17.2	5.3	9.6	8.1	13.6	19.4	56.1	**43.9**	.000
Evaluate plan of care	14.5	12.6	15.3	6.0	7.6	10.6	11.5	22.0	48.9	**51.1**	.000
Monitoring/Surveillance											
Monitoring patient physiological status	20.5	6.3	17.7	4.5	11.0	7.0	15.5	17.5	64.7	**35.3**	.000
Monitoring patient affect and behaviour	14.0	12.8	12.9	9.4	9.4	8.6	12.4	20.5	48.7	**51.3**	.000
Monitoring patient physical safety	18.1	8.7	15.4	6.8	8.5	9.5	13.7	19.3	58.8	**44.2**	.000
Follow‐up on change in patient status	15.8	10.9	15.7	6.7	9.2	8.8	12.6	20.4	53.2	**46.8**	.000
Review documentation by care team	14.1	12.6	14.4	8.0	8.3	9.6	9.8	23.1	46.7	**53.3**	.000
Provide adequate supervision of delegated tasks	14.4	12.4	11.3	11.1	7.8	10.1	12.8	20.1	46.3	**53.7**	.000
Hygiene											
Routine hygiene for patients	18.7	8.1	18.2	4.1	8.8	9.2	16.6	16.3	62.3	**37.7**	.000
Routine skin care	20.1	6.7	18.7	3.5	8.8	19.2	18.2	14.7	65.8	**34.2**	.000
Change soiled linen	16.3	10.6	17.7	4.4	9.0	9.0	11.9	21.1	54.9	**45.1**	.000
Nutrition											
Assist with feeding patient	19.1	7.7	17.5	4.9	11.3	6.7	17.7	15.2	65.6	**34.4**	.000
Administer enteral or parenteral nutrition	22.7	4.1	20.8	1.5	12.0	5.9	20.5	12.4	76.1	**23.9**	.000
Implement measures to promote physical comfort	19.5	7.3	14.8	7.5	9.2	8.8	13.9	19.0	57.4	**42.6**	.000
Mobility											
Assist with needed ambulation	17.2	9.6	14.6	7.6	9.4	8.6	13.3	19.7	54.4	**45.6**	.000
Mobilize or change patient position	18.1	8.7	16.9	5.3	8.7	9.3	13.5	19.5	57.2	**42.8**	.000
Assistance with bowel or bladder elimination	18.4	8.4	17.6	4.6	10.2	7.8	17.3	15.7	63.5	**36.5**	.000
Treatment, Tests, Procedures											
Administer medications	22.4	4.3	20.6	2.3	12.6	5.4	21.4	11.6	76.4	**23.6**	.000
Change intravenous access sites, tubing or dressing	21.4	6.0	16.7	3.7	10.9	7.5	17.6	16.1	66.7	**33.3**	.000
Provide wound care	20.5	6.3	19.7	2.6	11.8	6.0	18.0	15.0	70.0	**30.0**	.000
Prepare patients for treatments, tests or procedures	20.5	6.3	18.9	3.5	8.4	9.6	15.3	17.5	63.1	**36.9**	.000
Adherence to safe patient handling guidelines	17.1	9.6	13.9	8.6	9.4	8.6	13.3	19.6	53.7	**46.3**	.000
Patient and family teaching	14.1	12.6	13.6	8.7	7.1	10.9	11.2	21.7	46.0	**54.0**	.000
Emotional or psychological support	13.1	13.7	10.5	11.9	7.0	10.8	7.8	25.2	38.4	**61.6**	.000
Adhere to infection control guidelines	21.7	5.0	19.3	3.1	9.6	8.3	16.3	16.6	66.9	**33.1**	.000
Timely response to request/need	11.3	15.5	8.6	13.8	6.0	11.9	13.1	19.9	30.0	**61.0**	.000
Coordination and Discharge Planning											
Important conversation with patient or family about discharge	14.8	12.1	14.6	7.6	6.4	11.5	14.9	18.1	50.8	**49.2**	.000
Important conversation with team member	13.0	13.7	13.1	9.2	6.5	18.0	11.3	21.6	44.0	**56.0**	.000
Important conversation with external agency	17.2	9.6	17.0	5.2	8.8	9.2	16.8	16.2	59.8	**40.2**	.000

^†^ Boldface text indicates percentage of nurses from participating countries with a frequency rating higher than never (percentages are counted from the whole sample)

### 5.3 Factors contributing to unfinished nursing care

Unfinished care correlated significantly with overall job satisfaction, nurse‐assessed quality of patient care on unit, intention to leave the actual workplace and perceived adequacy of staffing. Correlations were low and moderate (Table 4).

**Table 4:** Correlations between unfinished nursing care (PIRNCA score), job satisfaction, nurse‐assessed quality of patient care on unit, perception of staffing adequacy, intention to leave the workplace, intention to leave profession, age and years of experience

	Years of experience (total)	Years of experience‐ workplace	Intention to leave workplace	Intention to leave profession	Perception of staffing adequacy	Unfinished care	Quality of care	Job satisfaction
Age	0.946**	0.697**	−0.214**	0.016	0.021	−0.037	0.017	0.097**
Job satisfaction	0.075**	0.002	−0.479**	−0.236**	−0.205**	−0.423**	0.541**	
Quality of care	0.066*	0.031	−0.223**	−0.130**	−0.231**	−0.438**		
Unfinished care	−0.024	0.049	0.302**	0.115**	0.122**			
Perception of staffing adequacy	−0.118**	−0.067*	0.089**	−0.009				
Intention to leave profession	0.021	0.071*	0.462**					
Intention to leave workplace	−0.182**	−0.082**						
Years of experience (workplace)	0.737**							

* *p* < .05; **p < .01; ***p < .001.

To establish the relationship between nurse, hospital and unit variables and unfinished nursing care, stepwise multiple regression analyses were performed (variables were selected according to the results of correlation analyses). Unfinished care was predicted by six variables (Table 5), explaining a total of 31% of the variance. Type of hospital and nurse‐assessed quality of patient care significantly predicted unfinished nursing care. The higher the nurse‐assessed quality of patient care reported, the less significant the extent of nursing care left unfinished.

**Table 5:** Regression of the PIRNCA

Predictor	*R*	*R* ^2^‐change	*B*	*T*	*p*
Unfinished nursing care	(*F*_total_ (19.42) = 69.08; *p* < .000)				
Intention to leave workplace	0.239	0.057	−0.082	−2.763	.006
Perception of staffing adequacy	0.279	0.021	0.099	3.460	.001
Type of hospital	0.409	0.089	−0.205	−7.234	.000
Type of unit	0.456	0.040	−0.180	−6.475	.000
Quality of care	0.531	0.075	−0.199	−6.161	.000
Job satisfaction	0.552	0.023	−0.199	−5.552	.000
Constant			24.26		

Abbreviations: *b*, Beta standardised regression coefficients; *F*_total_, *F* test of overall significance; *R* ^2^‐change, change in *R* ^2^; *R*, correlation coefficient; *T*, *t* statistic.

Nurses from university hospitals reported more unfinished care than nurses from general hospitals. In addition, nurses reporting an intention to leave their workplace reported more unfinished care than nurses with no intention of leaving (Table 6).

**Table 6:** Variables (nurse, unit and hospital characteristics) of the unfinished nursing care

Variable	Mean square	*F*	*p*	Partial Eta squared
Unfinished nursing care (PIRNCA score)				
**Education**				
Secondary nursing school or diploma/Bachelor degree or higher	0.009	0.029	.864	0.000
**Unit**				
Medical units/surgical units/ICU/others	0.773	2.406	.066	0.008
**Hospital type**				
University/faculty/teaching/general	2.684	8.350	.004	0.010
**Hospital size**				
<300 beds/300–600 beds/>600 beds	0.068	0.213	.808	0.000
**Leaving intentions**				
thoughts to leave actual workplace/no leaving intentions	2.126	6.614	.010	0.008

## 6 DISCUSSION

This is the first multinational study describing and comparing the prevalence of unfinished nursing care across Central European countries. Compared to the growth of research concerning unfinished care in the Western European context, or in the USA and Australia, only a small number of papers have examined the prevalence of unfinished care in relatively new member states of the European Union.

Overall, in line with results of previous multinational studies investigating unfinished care, high between‐country variations in unfinished care were found in this study. In addition, consistent with previous reviews (Jones et al., 2015; Papastavrou, Andreou, & Efstathiou, 2014b) and multinational quantitative studies (Ausserhofer et al., 2014; Blackman et al., 2018), the current study confirmed a high prevalence of unfinished nursing care in central European countries. In their review, Jones et al. (2015) emphasized that prevalence of unfinished care based on composite mean scores ranged only from “rarely” to “sometimes,” while prevalence based on the percentage of positive responses was higher. These results have been replicated in this study. The estimate of unfinished care based on composite mean score was approximately “rarely,” indicating low prevalence. However, the percentage of positive responses of nurses (answering higher than “never”) demonstrated higher overall prevalence of unfinished nursing care. The most frequently unfinished care interventions observed in the study were in line with results from previous studies with the most frequently unfinished care activities were related to surveillance (Jones et al., 2015). All of the most frequently unfinished care activities identified in this study were among the “top five” most frequently left unfinished (Jones et al., 2015)—timeliness of care; coordination and discharge planning, emotional or psychological support, patient education, monitoring/surveillance and supervision of care. The most often unfinished care activity of Czech and Slovak nurses was a timely response to patient requests. The most often unfinished care activity of Polish nurses was patient and family education. The most often unfinished care activity of Croatian nurses was emotional or psychological support. According to Bragadóttir and Kalisch (2018), the “different roles and responsibilities of nurses, based on their education and training, may cause them to have different mental models as to the priority of various elements of nursing care.” Such mental models may affect their responses and their decisions about nursing activities which can be postponed. The dominance of a biomedical model of care in all surveyed countries might influenced our results. A biomedical model together with technology in acute care departments may overshadow holistic and humanistic care (Mazzotta, 2016).

The high level of unfinished care reported by nurses from university hospitals in this study is an important finding. Similar results were reported in an Icelandic study (Bragadóttir, Kalisch, & Tryggvadóttir, 2017). University hospitals have a unique role, as they are training, educational and professional medical centres. They provide a complete range of medical services and cooperate in the training of medical, nursing and allied healthcare professionals. Our results direct attention to the need to recognize this dual role of university hospitals, as well as the multiple demands on nurses involved in the process of clinical education.

It would seem that several fundamental nursing tasks are regularly omitted across countries, although local findings from different countries hint that the extent of and kinds of activities unfinished may vary across countries (e.g. Blackman et al., 2015; Jones et al., 2015; Palese et al., 2015; Papastavrou, Andreou, Tsangari, et al., 2014a). Several other studies have compared the prevalence of unfinished nursing care across countries (e.g. Ausserhofer et al., 2014; Blackman et al., 2018; Kalisch, Doumit, Lee, & Zein, 2013; Kalisch, Gosseline, & Choi, 2012). These studies generally reported high between‐country differences in the level of nursing tasks that were unfinished. A comparison of unfinished nursing care across Australia, Cyprus and Italy found that country influenced the amount of unfinished nursing care (Blackman et al., 2018). We can only hypothesize about the reasons for the differences in unfinished care between countries. One possibility might be the various types of hospital in the countries included. The Polish and Croation samples included nurses from university hospitals (potentially with dual roles), and in these cases, more unfinished nursing care were reported. The Czech and Slovak samples included nurses from both university as well as private hospitals. The clinical significance of differences between countries remains undetermined (Jones et al., 2015).

In a previous study using the MISSCARE survey, Czech and Slovak nurses reported a significant amount of unfinished nursing care (Zeleníková, Gurková, & Jarošová, 2019). Nurses from Slovakia reported more unfinished care than nurses from the Czech Republic. These results are consistent with those of the present study. Comparable studies have not previously been carried out in either Croatia or Poland, where unfinished nursing care was thus being studied for the first time.

The instrument used for measuring unfinished care, the PIRNCA, was developed in the USA and has not been used (translated and validated) in European countries before. European countries have, for the most part, used the BERNCA (Dhaini et al., 2017; Papastavrou, Andreou, Tsangari, et al., 2014a; Schubert et al., 2008; Zúñiga et al., 2015) or MISSCARE Surveys (Ausserhofer et al., 2014; Bragadóttir, Kalisch, Smáradóttir, & Jónsdóttir, 2015; Bragadóttir et al., 2017; Palese et al., 2015; Papastavrou, Charalambous, Vryonides, Eleftheriou, & Merkouris, 2016; Sist et al., 2017; Zeleníková et al., 2019). This is the first European study reporting results of unfinished care measured using the PIRNCA. All national versions of the PIRNCA (Croatian, Czech, Polish and Slovak) showed good reliability and unidimensional factor structure and can be used for future research. The instrument can also be used by managers for evaluating care, organizational characteristics and safety issues in these countries.

The lowest overall PIRNCA score was seen in the Czech sample. This can be explained by the fact that, of the four countries investigated, the Czech Republic had the highest number of nurses/per 1,000 people. On the other hand, the highest overall PIRNCA score was seen in the Croatian sample—Croatia having the lowest number of nurses/per 1,000 people. To a certain extent, this points to staffing (an employment characteristic) as one of the important predictors of unfinished care. In addition, in our study, unfinished care was predicted by six variables: intention to leave workplace, perceived adequacy of staffing, type of hospital, type of unit, quality of care and job satisfaction, all of which are employment or organizational characteristics. Although all these variables are important components of unfinished nursing care, they do not constitute the entire problem. A lower percentage of variance indicates that there are other variables that might affect and predict unfinished care. In Ausserhofer et al. (2014), organizational factors were found to influence unfinished nursing care. In a Swiss study (Zúñiga et al., 2015), nurses reported better quality of care when the amount of unfinished care was lower. Jones et al. (2015), in their review, found the strongest association to be between perceived adequacy of staffing and unfinished care. The negative impact of unfinished care on intention to leave, and on job satisfaction, has been reported by the authors of several reviews (Jones et al., 2015; Papastavrou, Andreou, & Efstathiou, 2014b). There are also serious concerns about job satisfaction as a key contributor to quality of care and nurse turnover (Lu, Zhao, & While, 2019). Further research is needed to fully understand the association between unfinished nursing care, job satisfaction, intention to leave and quality of care.

## 7 LIMITATIONS OF THE STUDY

The study has several limitations. The first is that it used an instrument which was not developed for the target languages or cultures and developer of the instrument was not included in back translation process. Content validity was not evaluated. The second limitation is its use of a non‐randomized sample of nurses. The third limitation is its use of single‐item questions for measuring overall job satisfaction, quality of care and intention to leave. The final limitation is the use of a cross‐sectional study design, which did not allow us to arrive at firm conclusions regarding causality of predictors.

## 8 CONCLUSION

The research results confirmed a high prevalence of unfinished nursing care in the four selected European countries (Croatia, the Czech Republic, Poland and Slovakia). In this study, type of hospital and quality of care most significantly predicted the prevalence of unfinished care.

## 9 IMPLICATIONS FOR NURSING MANAGEMENT

The results of this study point to certain aspects of nursing care which are more frequently left unfinished than others. Most of the unfinished nursing care tasks were time‐consuming nursing activities. Its findings have implications for nurse managers. The significance of prioritizing basic nursing care is obvious, and our results indicate the nursing care activities that nurse managers need to address. Since employment and organizational characteristics were among the predictors of unfinished care, nurse managers can concentrate on how to manage these predictors, which can influence the quality of care provided to patients. Improving staffing as well as using technologies could be effective in preventing unfinished nursing care.

## 10 ETHICAL CONSIDERATIONS

Permission to conduct the study was received from the CORE group of COST Action RANCARE. Ethical committee approval was obtained from Ethical committee of Medical Faculty University of Ostrava, Czech Republic (no. 15/2018), as a main coordinator of the study as well as from Ethical committees of all four Croatian university hospitals included in the study. Institutional approvals were also obtained before conducting research. All participants were fully informed about the purpose of the study, voluntary participation, their anonymity and confidentiality. Answering the survey was seen as voluntary consent to participate. At any time, participants had the right to withdraw. Confidentiality of the participants was respected.

## AUTHOR CONTRIBUTIONS

RZ, EP, EG, IU, AF, IP, DJ and KZ made substantial contributions to conception and design, or acquisition of data, or analysis and interpretation of data; given final approval of the version to be published; each author should have participated sufficiently in the work to take public responsibility for appropriate portions of the content; and agreed to be accountable for all aspects of the work in ensuring that questions related to the accuracy or integrity of any part of the work are appropriately investigated and resolved; RZ, EG, EP, IU and AF involved in drafting the manuscript or revising it critically for important intellectual content.
